# Recent Updates on Corticosteroid-Induced Neuropsychiatric Disorders and Theranostic Advancements through Gene Editing Tools

**DOI:** 10.3390/diagnostics13030337

**Published:** 2023-01-17

**Authors:** Manisha Singh, Vinayak Agarwal, Divya Jindal, Pranav Pancham, Shriya Agarwal, Shalini Mani, Raj Kumar Tiwari, Koushik Das, Badrah S. Alghamdi, Tukri S. Abujamel, Ghulam Md. Ashraf, Saurabh Kumar Jha

**Affiliations:** 1 Department of Biotechnology, Jaypee Institute of Information Technology (JIIT), Noida 201309, India; 2 Department of Molecular Sciences, Macquarie University, Macquarie Park, NSW 2109, Australia; 3 School of Health Sciences, Pharmaceutical Sciences, UPES, Dehradun 248007, India; 4 Department of Physiology, Neuroscience Unit, Faculty of Medicine, King Abdulaziz University, Jeddah 21589, Saudi Arabia; 5 Pre-Clinical Research Unit, King Fahd Medical Research Centre, King Abdulaziz University, Jeddah 21589, Saudi Arabia; 6 King Fahd Medical Research Center, King Abdulaziz University, Jeddah 21589, Saudi Arabia; 7 Department of Medical Laboratory Sciences, Faculty of Applied Medical Sciences, King Abdulaziz University, Jeddah 21589, Saudi Arabia; 8 Department of Medical Laboratory Sciences, College of Health Sciences, University of Sharjah, University City, Sharjah 27272, United Arab Emirates; 9 Department of Biotechnology, School of Engineering & Technology, Sharda University, Greater Noida 201310, India; 10 Department of Biotechnology, School of Applied & Life Sciences (SALS), Uttaranchal University, Dehradun 248007, India; 11 Department of Biotechnology Engineering and Food Technology, Chandigarh University, Mohali 140413, India

**Keywords:** genetic variants, epigenetic factors, major depressive disorders (MDD), cognitive disorders, genome-wide association studies (GWAS), whole exosome sequencing (WES)

## Abstract

The vast use of corticosteroids (CCSs) globally has led to an increase in CCS-induced neuropsychiatric disorders (NPDs), a very common manifestation in patients after CCS consumption. These neuropsychiatric disorders range from depression, insomnia, and bipolar disorders to panic attacks, overt psychosis, and many other cognitive changes in such subjects. Though their therapeutic importance in treating and improving many clinical symptoms overrides the complications that arise after their consumption, still, there has been an alarming rise in NPD cases in recent years, and they are seen as the greatest public health challenge globally; therefore, these potential side effects cannot be ignored. It has also been observed that many of the neuronal functional activities are regulated and controlled by genomic variants with epigenetic factors (DNA methylation, non-coding RNA, and histone modeling, etc.), and any alterations in these regulatory mechanisms affect normal cerebral development and functioning. This study explores a general overview of emerging concerns of CCS-induced NPDs, the effective molecular biology approaches that can revitalize NPD therapy in an extremely specialized, reliable, and effective manner, and the possible gene-editing-based therapeutic strategies to either prevent or cure NPDs in the future.

## 1. Introduction

Corticosteroids (CCSs) have been used to treat a variety of inflammatory conditions as an immunosuppressive medication for decades. However, many CCSs, including prednisone, methylprednisolone, dexamethasone, and adreno corticotropic, have been shown to have adverse psychiatric consequences [1]. Despite being an efficacious and resolute drug for anti-inflammatory reactions, it is also an immunosuppressive medicine; however, its frequent use globally has also led to an increase in mental health issues and concerns [2]. Corticosteroids inhibit the synthesis of inflammatory proteins while increasing the release of anti-inflammatory ones by various signaling pathways such as nuclear factor kappa B (NF—ƙB), mitogen-activated protein kinase (MAP kinase), etc., but while doing so, they cause other concerns such as immunosuppression, osteoporosis, glaucoma, hypertension, growth retardation, etc., [3].

It was further confirmed by Wolkowitz et al. (2009) that increased levels of CCSs, particularly glucocorticoids (GC), induce the occurrence of psychiatric symptoms. It has also been proposed that stress-induced hypothalamic–pituitary–adrenal axis activation accelerates psychosomatic symptoms by altering neurotransmitter levels in cortical regions [4].

Steroid-induced psychiatric symptoms are diverse and range from mild to severe forms involving behavioral, affective, and cognitive regions in the brain [5]. Although the current causative mechanism is not known, there is evidence of significantly reduced immunoreactivity to corticotropin, norepinephrine, and beta-endorphin affecting the hippocampus and amygdala regions of the cerebral cortex [6]. Moreover, to date, there is no registered drug available for the treatment of CCC-induced NPDs.

Psychiatric symptoms brought on by steroids replicate and induce metabolic, neurologic, and cardiovascular complications. In addition to this, steroids induce withdrawal symptoms, mood disorders, paraneoplastic syndrome, bipolar disorder, etc., in affected patients. While NPDs are on the rise globally, due to extrinsic and intrinsic factors, the prospect of an additional patient add-on load due to CCS usage significantly raises the alarm, and is a vexing concern. Therefore, better and more targeted therapeutic approaches are needed in this direction [7,8,9,10]. In the last two decades, the introduction of genetic medicine, “Gene editing” or “Gene therapy”, has revolutionized the medical field with its fascinating concepts and personalized approaches. The genetic medicine concept was initiated when oligonucleotide-based therapies provided therapeutic relief for many diverse pathologies (cancer, neuromuscular diseases, hemophilia 1, etc.) [11], followed by adeno-associated virus (AAV) gene transfer to CNS [12] and now the CRISPR/Cas9 approach. The advent of the CRISPR approach is reported to be a targeted therapeutic approach for neuropsychiatric forms such as seizures, autism, and cognitive decline. The pathological pathways of NPDs are so complicated that they further upset the balance between excitation and inhibition [13] mechanisms and are likely to be of multifactorial descent, encompassing both genetic predisposition and environmental factors [14,15]. Also, many reports suggest a deeper genetic involvement in conditions such as depression, obsessive-compulsive disorders, etc., and in such conditions gene therapy seems to fit perfectly by disrupting the causative genes [16]. As mentioned earlier, the rate of such neuropsychiatric complications is relatively higher in subjects administered CCSs, a synthetic analog of the natural steroidal compounds synthesized by the adrenal cortex in humans [17]. It has been recounted that patients on CCSs for more than 10–14 weeks very frequently exhibit complications of mania (27.8%), psychosis (13.9%), delirium (10.1%), and depression (40.5%) [18]. In such cases, gene therapy can play a pivotal role in better prognosis [19]. This review discusses the conditions where CCSs are used as a drug therapy followed by a rise in neuropsychiatric disorders due to its continuous use, and the possibilities of gene therapy interventions including CRISPR Cas-9 approaches in exterminating such complications. It has been reported that CCSs play a vital role in the moderation or regulation of gene expression in the CNS [20]. The brain effectively expresses two types of corticosteroid, glucocorticoids (GR) and mineralocorticoids (MR), and both differ from each other in their distribution profile and affinity [21,22]. However, specific receptor molecules are equally important for the efficient facilitation of their expression, so in human physiology MR and GR receptors have been widely identified as the same [23]. Both corticoid receptors exist in CNSs and are responsible for the mediation of several genomic as well as non–genomic neural activities [24]. Glucocorticoids and MRs are ligand–driven transcription factors that tend to exist in their native condition of unbound state in the cytosolic regions [25], and their gene regulation is primarily regulated by the gene response elements specific to GRs [26]. Also, broad-spectrum expressions are specially regulated by GR rather than MR and they efficiently modulate their specific genomic as well as non–genomic mechanisms [27,28]. There are various ways of facilitating or influencing gene regulation, and although polymorphism has not been approved as a conventional gene regulatory mechanism yet, it has been observed as being extremely influential in doing so. Polymorphism at a specific GR gene [NR3C1] has been identified to cause minor or major variations associated with GR functioning [29]. Further, the adverse effects associated with CCS administration are caused due to the transactivation of genes participating in biochemical and metabolic processes, and GR ligands tend to cause conformational changes to the GR/protein interaction [30]. On the other hand, they do not cause any changes in the GR/DNA binding-dependent mechanism resulting in selective GR agonists (SEGRAs) [31]. As discussed earlier, the genetic variations caused by GR are significantly higher than MR, triggering a path for the manifestation of several cognitive dysfunctions and psychosis phenomena [32], thus necessitating the need for a genetic intervention that can resolve the genetic alterations associated with inter- and intra-subject variability in response to GR- and steroid-related toxicity.

In addition, the genome-engineering tool CRISPR/Cas 9 has pioneered several genome and epigenome alteration targets all at once [33]. The CRISPR screens are specifically stronger when combined with a pluripotent stem cell technique, ensuring the sourcing of differentiated cells such as neurons, glial cells, and brain organoids from diseased subjects [34]. The CRISPR tool and its associated protein (Cas) system consist of a CRISPR locus (which has 2–375 repeat sequences and 21–48 bp, 1–374 interspersed spacer sequences (26–72 bp)) as well as Cas genes. This entire system is used in different cell line models (human isolated pluripotent stem cells (iPSCs)) and animal models to sequence the therapeutic approach in various NDDs by knocking out and repairing the mutant genes [34]. The most significant hereditary disorders are anxiety disorders (AD), schizophrenia (SP), major depressive disorders (MDD), bipolar disorders (BP), autistic spectrum disorders (ASD), and attention deficit hyperactivity disorders (ADHD) [35]. There are many reported faulty genes responsible for these conditions, including AS3MT, CSMD1, ANK3, CNNM2, TENM4, CACNA1C, PPP1R11, CACNB2, NT5C2, DPR1, TCF4, ITIH3, SYNE1, TRIM26, and ZNRD1, mainly expressed in neuronal activity, immune regulation, synaptic transmission, and cellular mechanism linked to the above-mentioned disorders [36]. These can be subjected to CRISPR for resolving the genetic alterations in the affected subjects.

## 2. Biogenesis and Expression of Corticosteroids in the Brain

Corticosteroids are the kinds of steroidal hormones that are either produced by the body or are artificially synthesized [37]. The biosynthesis of these hormones occurs from cholesterol within the adrenal cortex site, which is also known as the primary site of several steroidogenic biosynthetic reactions [38]. Mostly, such manifestations are caused by the ultradian or circadian variations and external artificial administration, or are elicited in response to a certain stressful stimulus [39]. The mechanism of action for both GR and MR highly overlaps due to their shared and almost identical DNA-binding domains, yet their expression significantly differs as genes regulated by them are not common [40]. The 21 hydroxylase and 17 α-hydroxylase enzymes are strongly associated with the synthesis of CCS hormones in the human system [41] and P450c17 is the only enzyme mediating the activity of both 17,20-hydroxlyase and 17 α -hydroxylase (steroid 17 alpha-monooxygenase, EC 1.14.99.9) during the synthesis of steroid hormones [42]. Then, there are P450c17 isozymes that are proposed to mediate the same in the testis and adrenal glands. Next, pregnenolone is transformed into mineralocorticoids because the adrenal zona glomerulosa lacks P450c17 activities and the same pregnenolone is transformed into glucocorticoid cortisol in the zona fasciculata because there is a presence of active 17-hydroxylase [43,44]. Both these processes take place in the zona reticularis, where pregnenolone is transformed into sex steroids. Electron transport is the main mechanism controlling the 17, 20-lyase process from NADPH via POR (P450-oxidoreductase) [45].

The CCS expression profile also exhibits altered cellular mechanics in response to stress levels and is extensively influenced by CCS and stress hormone interplay along with the effects facilitated by GR and MR [46]. Such expressional interplaying response systems further add to complexity at the cellular level. Several prior research studies have suggested that only a balanced response, calibrated between the two systems, can potentially lead to resilience. This balance can be hampered by several distinct conditions such as unremitting exposure to stress, especially in genetically vulnerable individuals, which can cause the aggravated manifestation of the pathology, and such cases are not only restricted to psychiatric disorders but instead extend to several neurological pathologies [47].

Another perspective for the involvement of CCSs in initiating the progression of NPDs is the perfusion of MR and GR in the neuronal cells of the hippocampus under the limbic system, confirmed further by various diagnostic studies in autoradiography, in situ hybridization, and radioligand-binding analysis [48], since a hippocampus region in CNS is directly involved with mood and stress response, controlled by the hypothalamic–pituitary–adrenal (HPA) axis [49]. It has also been noted that the limbic system supports interaction between GR and MR, wherein MR exhibits a higher affinity towards corticosterone (CORT) and aldosterone (ALDO) binding [50], though its perfusion at the CORT site is 2–3 fold higher in comparison to the general receptor concentration circulating in the CNS. However, GR expresses 6–10 fold lower affinity than MR, [27,51].

### 2.1. Gene Regulation by Glucocorticoids (GR)

Corticosteroids are sub-classified as one of the steroidal molecules and thus, GR also belongs to the nuclear hormone receptor superfamily; thus, such receptors exhibit distinct expression over ligand-activated transcription factors [52]. The glucocorticoid is constituted of three chief modular structural domains: N-Terminal Domain (NTD), Ligand Binding Domain (LBD), and DNA Binding Domain (DBD), and these domains further facilitate their expression independently [53]. The NTD is known to be highly influential in the transcriptional machinery as it contains several transactivation regions such as AF1 (Activation Factor 1) / tau1/enh2 [54,55]. Moreover, the AF1 region acquires folded conformation that under specific physiological conditions binds to the GR response element (GRE), resulting in the triggering of gene regulation (Reddy et. Al) [56], and this alteration in the genetic sequence of GRE initiates the conversion of the co-repressor to a co-activator expression. Xavier et al. (2016), in their study, observed that co-repressor protein GRIP 1 (Glutamate receptor-interacting protein 1) molecules increase their co-activator properties when they bind with GR, located on specific GRE [57]. In addition to this, GR co-activator complex models are also responsible for recruiting additional co-activator molecules to the assembly [58].

### 2.2. Gene Regulation by Mineralo Corticosteroids (MR)

One of the decisive roles in MR response is the moderation and regulation of MR expression levels [46]. It exerts its expression in the hippocampal region for balancing functional action. The hormones in the CNS such as progesterone, as well as serotonin, control the MR mRNA expression [59]. Additionally, it has been observed that after interacting or binding to a ligand molecule, the MR travels to the nucleus and initiates itself to function as a transcription factor. The MR has firm binding specifically to the Human Response Element (HRE), located at 10 kb downstream/upstream from the transcriptional site [60]. Some studies have categorized MR as a ubiquitin transcription factor. Its real-time PCR quantification presents various interesting anatomical expressions that show MR regulation elevating its expression in CNS rather than GR [61]. In humans, aldosterone is identified as the most important natural ligand to the MR, besides cortisol that aids in facilitating MR’s expression at the messenger or at the protein level in cells where either of these two ligands have been found to regulate the process [62]. Furthermore, it has been reported that epithelial expressions of MR have always been associated with a simultaneous expression of 11 β-hydroxy steroid dehydrogenase 2 (11β-HSD2), permitting the aldosterone to selectively activate the MR, by primarily converting the GR hormones to their 11-keto analogs [63,64].

## 3. Non-Genomic Effects of Corticosteroids (CCSs) Related to CNS

The actions of CCSs (Groeneweg et al., 2011), further stated as the facilitation of memory consolidation, is dependent on gene transcription through activation of the genomic GR and MR [65], though CCSs also affect behavior and memory in a rapid and presumably non-genomic manner. Therefore, the rapid effects of CCSs have been described for several adaptive behaviors, including rapid facilitation of novelty-induced locomotion, context-dependent aggression, and risk assessment behavior [66]. Interestingly, MR is repeatedly reported to get involved in such behavior. As these behavioral effects are rapidly induced by stress doses of corticosterone, they always seemed incompatible with the constitutively active genomic MR [67]. Moreover, the lower affinity towards membrane-MR could prove to be the logical basis for these effects [68]. In behavioral studies related to the regulation of memory, it has been found that GR has a predominant function in memory consolidation, while MR is mostly involved in memory retrieval and learning strategies [69]. A similar convergence of functions is seen in the rapid facilitation of memory consolidation by corticosterone that depends on (presumably membrane-localized) GR in the cortex [69,70]. The application of antagonists for endocannabinoid signaling in the amygdala blocks the corticosterone-induced effects on memory consolidation [71]. Together, this suggests that the membrane-GR-mediated and endocannabinoid-dependent inhibition of neuronal excitability might be implicated in memory consolidation [72]. In contrast, corticosterone’s effects on memory retrieval seem to be MR-mediated.

**Figure 1:**
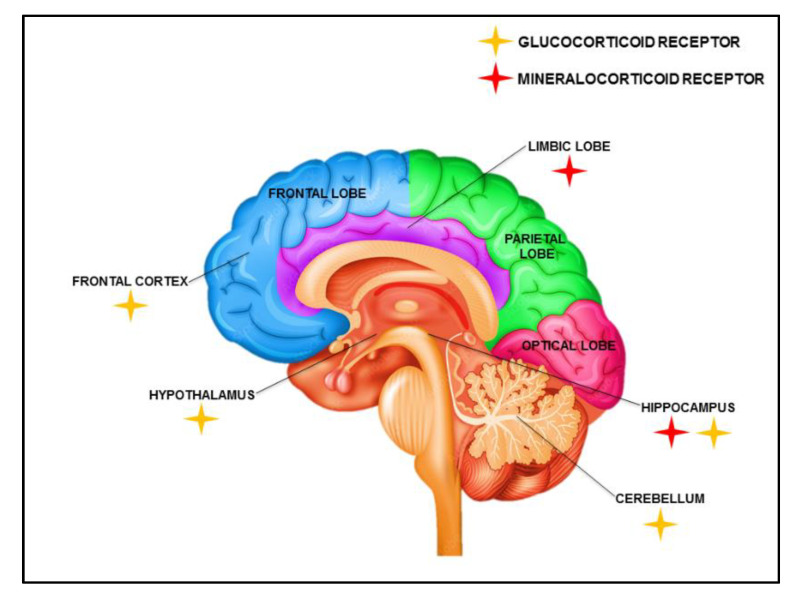
Illustrating distribution and existence of glucocorticoids (GR) and mineralocorticoids (MR) in the central nervous system.

The GR consolidates through both rapid and delayed (genomic) pathways [73], whereas MR has a specific (non-genomic) role during memory retrieval, possibly as a mechanism to focus attention on new stress. Taken together, in its role as a rapid corticosteroid sensor, the MR facilitates adaptive behavior in context to stressors while inhibiting behaviors that are no longer relevant [74]. The distribution of GR and MR in cerebral cortex regions is shown in Figure 1.

## 4. Therapeutic Action of Corticosteroids

Corticosteroids have shown an immense application in therapeutics, based on their immunosuppressive effects by modulating immune functions, and are generally used for allergic reactions and inflammatory diseases [75]. They show adverse side effects when consumed in higher doses over a longer period, and suppress the pituitary–adrenal axis [76].

They have always been considered molecules with an ability to elicit immune-modulatory effects and inflammatory responses including delayed-type hypersensitivity responses, mediated by immune cells (macrophages and T-cells) [77]. Their mechanism of action is observed at multiple levels, as initially they decrease the circulation of T-cell precursors and macrophages available with reduced Interleukin-1 and 2 productions [2]. Other responses are mostly related to immunoglobulin (Ig) and antibody production, aided by lower levels of CCS, and subsequently lowered serum content in Igs [78]. Corticosteroids are mainly involved in the interaction with cellular receptors leading to modification of DNA and the transcription process, eventually down-regulating and inhibiting various immunity-triggering responses such as cell-mediated immunity and inflammatory cell aggregation [79]. They also suppress the expression of endothelial leukocyte adhesion molecule I, and intracellular and vascular adhesion molecules (ICAM and VCAM-1), that in turn oppose leukocyte trafficking [80]. Apart from this, CCSs are involved in attenuating a rise in fever by decreasing the production of macrophages, dendritic cells, and monocytes. Moreover, GRs are reported to reduce the chances of morbidity in major pulmonary diseases (asthma, croup, respiratory distress syndrome (RDS), reactive airway diseases, chronic obstructive pulmonary diseases, and respiratory infectious disorders) by restoring pulmonary functions [81]. For such pulmonary disorders, inhalation-type CCS preparations are recommended that control and can be favorable for early response to an allergen with prolonged treatment, severe pulmonary conditions and airway hyperresponsiveness instantly. They directly employ inhibitory effects on inflammatory cells through genomic systems and mechanisms [82]. Corticosteroids can be administered in combination using the synergistic inhibition properties of β 2-agonist [83] and by increased expression of β-2 adrenergic receptors through increased gene transcription [84]. In the case of allergens (spores or bacterial sources), their associated reactions are controlled efficiently by CCSs [85]. Meta-analysis of RID demonstrated improvements in people administered with systemic (dexamethasone) or nebulized (budesonide, dipropionate, flunisolide) corticosteroids [86].

Moreover, for auto-immune or inflammatory disorders of joints, intraarticular corticosteroid administration is recommended to avoid obstinate synovitis of joints/muscles and acts as an effective adjunct in osteoarthritis [87]. They are also effective in lupus manifestations, based on their major and minor organ involvement. The use of GR is mediated by genomic functions through binding to cytosolic glucocorticosteroid (GC) inducing and inhibiting the synthesis of proteins by transactivation and trans-repression machinery, respectively [88]. Due to their lipophilic nature, GRs pass through the cell membrane and bind to expressed cGR leading to genomic and non-genomic effects in cortical regions. During their genomic mechanisms, they interact with heat shock protein 90 (Hsp90) molecules and cGR with Src homology domains causing conformational changes [30]. Glucocorticoid complex with cGR is translocated to the nucleus for further action and Hsp90 molecules with Src homology are directed to the cGR-mediated non-genomic mechanism [89], whereas, in their non-genomic mechanism, GRs interact with membrane glucocorticosteroid (mGR) and integrate into the cell by mediating anti-inflammatory and immune-modulatory responses along with adverse effects [90].

## 5. Types of CCS-Induced NPDs

There are various categories of NPDs that are explicitly triggered by the use of CCSs, and the study by Savas et al., 2020 highlighted that after CCS consumption for a certain period, 2–4% of patients developed depression, anxiety, or became apathetic [91], while another 3% showed psychosis with hallucinations further confirming the trigger of schizophrenia. These adverse events were dose-dependent, time-dependent, or both and the remission resulted from suspension of the treatment or decreasing the dose of cortisone [5].

Similarly, CCSs reduced GABA neurotransmitter concentration, leading to anxiety, changes in mood, depression, seizures, and a decreased capacity to cope with chronic pain [92]. Corticosteroid-related disorders are more common in genetically vulnerable groups, and these observations support that a potential (genetic) predisposition in a particular subgroup may amplify after several major stressors early in life [38].

It has also been observed by Ciriaco et al., 2013, that there is a reduction in the execution of cognitive functioning in local and systemic CCS users [5], supporting the possibility of CCS effects on the brain. Although multiple studies have shown an association between corticosteroid use and other CNS disorders too, the pathophysiology of exogenous corticosteroid action on the brain is still not well understood, though prominent effects are noted selectively in cortical regions of the brain causing hippocampal atrophy, neuronal plasticity, neurotoxicity, and neurogenesis. Amongst all the other symptoms from CCS-induced NPDs, the most common ones are manic features including irritability, euphoria, pressured speech, hyperactivity, and distractibility [93]. Such patients also exhibit additional associated symptoms such as depression, hypomania, psychosis, panic attacks, agoraphobia, insomnia, catatonia, impaired memory, and obsessive-compulsive disorders (OCD) [93]. An extensive study conducted by Savas et al., 2020, in 83,592 adults (mean age 44 years, 59% women) of the general population, analyzed the relationship between corticosteroid use and a decline in cognitive functioning. The study indicated the CCSs that induced mood disorders were more prevalent in CCSs users in comparison to non-users, and also highlighted the different routes of administration with overall 70% single-type users, wherein systemically administered CCS use was only associated with a mood disorder, which was especially reflected in single-type users, whereas all other routes of administration (inhaled, dermal, nasal) also elicited a reduction in cognitive functioning but still comparatively lesser than the systemic form [91].

## 6. Associated Concerns after CCS Administration

CCSs are often known to cause psychiatric instabilities and cognitive defects such as cognitive impairment, memory loss, anxiety, hypomania, mood swings, insomnia, restlessness, etc., and show dependency traits on specific steroids [5,94]. Certain studies reported on the use of Prednisone (a commonly used CCS) causing NPDs in 1.3 % of patients with a daily dose of 40 mg, 4.6% when consuming a 41–80 mg dose, and an NPD incidence of 18.4% when consuming more than 80 mg per day. The study also revealed that the reduction in prednisone dose resulted in the decreased possibility of symptomatic signs in all classes [95]. Another study reported that the neuropsychiatric effects due to CCS administration range from 2 to 60% depending on its dose variation, course of therapy, and risk factors identified, such as genetic predisposition based on glucocorticoid receptor (GR) polymorphisms [96]. Additionally, catatonia has recently been linked with muscle stiffness, insomnia, and aberrant behaviors such as silence and stillness amongst a group of psychiatric symptoms, an outcome of chronic CCS administration [97]. Furthermore, a meta-analysis study performed with the subjects of prednisone therapy, with an average daily dose of 35 mg prednisone consumption, showed the occurrence of psychiatric symptoms two times more than those recorded in the placebo (*p* < 0.02) [98].

Currently, since there is more focus on the targeted delivery of CNS-based drugs, inhaled CCSs are widely administered. This surely helps in reducing the CCS dosage being given directly to the target site, but it also enhances the dearth of NPD incidences more commonly, with higher reported incidences of mood swings in exogenous corticosteroids with endogenous hypercortisolism [99]. There is also an evidence study that informs the development of a higher rate of irritability and insomnia in a five-year-old child following the consumption of budesonide (200µg/day) when treated for asthmatic symptoms; the symptoms reduced when the dose of budesonide was reduced [100]. Much similar research suggests that three quarters of children receiving steroid-based medication exhibit hyperactivity, irritability, sleeplessness, attention and memory concerns [101]. Prednisone is the most reported medication, followed by cortisone, dexamethasone, triamcinolone, betamethasone, and methylprednisolone and shows the highest level of side effects too, such as thyrotoxicosis, psoriasis, and mitral valve prolapse with ostentation, euphoria, etc. [102,103]. Therefore, the overall data available reveal that patients undertaking chronic and long-term steroidal therapy have developed increased episodes of depression, whereas acute steroidal therapy is concomitant with mania. Additionally, it has been particularly seen that cognitive deficits are the most prevalent consequence of CCS-administered treatment, irrespective of its administration for a longer or shorter time. It has been reported that short-term impairment happens due to progressive hippocampus neuron atrophy, leading to hippocampal dysfunction. Hall et al. reported a maximum number of cases for distractibility (79%), followed by intermittent memory impairment (71%), and a minimum number of cases for persistent memory impairment (7%), which eventually caused dementia [104].

Likewise, the psychic complications caused by CCSs are substance-induced mood disorders (with depressive, manic, and mixed features), substance-induced psychotic disorders, and delirium, but the mechanism for such disorders is not completely known [19]. Moreover, reduced levels of corticotrophin, norepinephrine, and ß -endorphin in cerebrospinal fluid (CSF) are directly linked to prednisone use, and further use of CCS also initiates an increase in glutamate release, and when it accumulates causes neuronal toxicity. The hypothalamic–pituitary–adrenal (HPA) axis, which retorts stress and controls the production of GR, is linked to depression, anxiety, declined cognitive functioning, and onset and recurrence of psychotic conditions [105]. According to research studies, GR antagonists targeting the HPA axis aid in the management of psychosis, depressive disorders, and cognitive dysfunction associated with these illnesses [7,106]. The Boston Collaborative Drug Surveillance Program found a 3% incidence of significant psychiatric effects among 718 hospitalized individuals receiving prednisone [107]. In cancer patients receiving higher levels of steroids, the prevalence of severe psychological impairment has been observed to vary from 5 to 10% [7].

## 7. Relevance of Gene Therapies in Eliminating CCS-Induced NPD

Gene therapy has shown pertinence for potential therapeutic intervention for the treatment of most genetically inherited diseases and, thus, has emerged as one of the most relied on therapeutic interventions in NPDs as well [108]. The gene-editing approach in the gene therapy category has proved to be more promising than the other subsets. This approach can be utilized for various purposes such as gene regulation/repair, total dormancy of toxic genes, etc., though its onsite efficacy and site-specific deliveries are unsolved queries to date leading to its deferred clinical application [109]. In traditional approaches, the defective genes were reinstated with the rectified ones through the various vector types and induced the new gene sets inside the cells to elicit the production of appropriate functional proteins as mentioned in Table 1 [110]. However, the concern comes when the entire amended gene sets cannot be accommodated into a vector type, as gene expression does have size restrictions and larger subsets are harder to arrange and deliver. The conventional forms of gene editing tools are favorable for autosomal recessive diseases, as they occur due to the exonic variants and are easily restored by fixing the correct copies of the target gene in the cells to start the normal functioning of site-specific proteins [111]. However, the actual problem persists when the same concept is applied to the autosomal dominant cases, where higher functioning is observed in exonic variants and hence, needs more extensive gene editing [112]. In addition to this, for fixing the protein functioning back, one needs the exogenous transfer of functional gene construct; also, the utilization of RNA interference and antisense oligonucleotides is needed for gene silencing along with more copied dose insertion to maintain efficacy [113]. Now, the most recent advancements in the last decades have improvised the entire gene-editing tool mechanism and extended its life of therapeutic effects by the CRISPR (Clustered Regularly Interspaced Short Palindromic Repeat) technique [114]. It has come up as a surprise gene-editing package to either edit a single or multiple gene targets, tested on a broader range of organisms.

## 8. Role of Non-Coding RNAs (ncRNAs)/mRNAs in NPDs

Non-coding RNAs (ncRNAs) are ones that are not translated to protein. The non-protein coding genes, on the other hand, are transcriptional sequeal along with most of them (80%) being transcriptionally operative and playing complex regulatory functions. Most of them are not classified as non-coding RNA genes and ncRNAs are divided into two categories depending on the size dimensions of their nucleotides: small ncRNAs (200 nucleotides (nt)) and long ncRNAs—lncRNAs (more than 200 nt). MicroRNAs (miRNAs), small interfering RNAs (siRNAs), piwi-interacting RNAs (piRNAs), small nucleolar RNAs (snoRNAs), and small nuclear RNAs are all examples of small RNAs (snRNAs). Further, mRNA transcription control, as well as substituting it with splicing and epigenetic modification changes such as RNA and chromatin alternatives, are also provided by them. These balancing acts are aimed at the neighboring transcripts (cis) or distant loci associated with their transcription (trans). Altogether, ncRNAs form a distinct stack of regulations in gene expression wherein they serve as important intermediate regulators in communicating information from genotype to phenotype asserts [135].

In the brain, a substantial majority of ncRNAs are widely expressed and their expression varies by brain area and cell type. Many research reports have highlighted that ncRNAs play an important role in brain evolution, development, homeostasis, stress response, and neuroplasticity [136]. Many NPDs, such as schizophrenia (SCZ), major depressive disorder (MDD), bipolar disorder (BD), and neurodegenerative disorders, are all impacted by the expression of ncRNAs inside the brain. Moreover, the ncRNA expression altercation impacts are not limited to the type of NPD but are more related to various regions in the brain [137]. 

Moreover, recently found endogenous closed-loop-structured ncRNAs known as circular RNAs (cirRNAs) are produced due to the back-splicing of protein-coding mRNAs during post-transcriptional processes. The findings of these investigations demonstrate that cirRNAs have the ability to regulate their respective miRNAs and their binding proteins. CirRNAs are extremely active at neural synapses and are more widely expressed in the brain than at any other loci, just as with miRNAs. With regard to their function and relevance to psychiatric disorders, miRNAs are the ncRNAs that have received the most attention. MiRNAs, which are typically 22 nucleotides long, are created by several enzymatic activities. Primary miRNA (pri-miRNA) is initially produced by the transcription of an encoded gene. The regulation of brain architecture and synaptic functioning is greatly influenced by several miRNAs [138,139,140,141,142].

## 9. Gene Editing of Target DNA Locus by CRISPR/Cas 9

**Figure 2:**
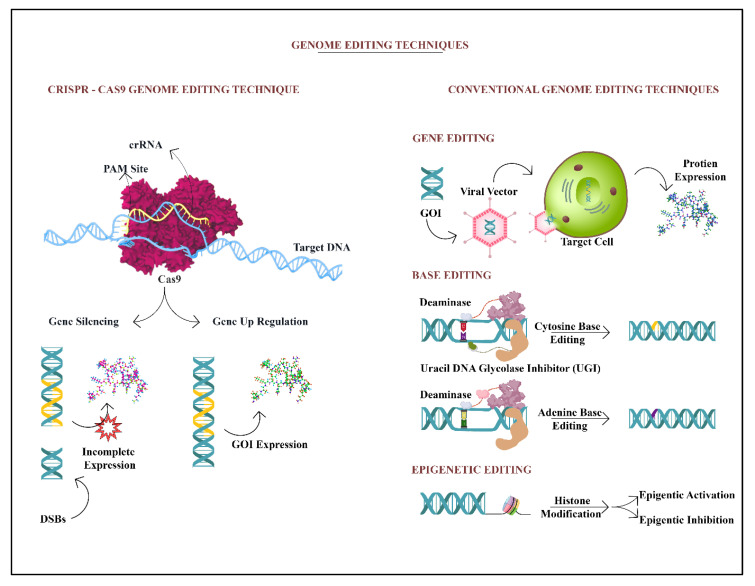
Schematic representation to exhibit the comparative method of gene editing by the most recently explored CRISPR/CAS-9 technique and the conventional tool for gene, base, and epigenetic editing.

The genome-engineering tools CRISPR/Cas have pioneered several genomes and epigenome alteration-targeting all at once. They were first discovered in bacterial species and Archaea as a crucial defense mechanism against invading viruses and plasmids [143]. When bacteria assimilate intruding DNA sequences within their genomes, these sequences are translated into CRISPR RNAs (crRNAs) that allow Cas endonucleases to target the invading DNA [144]. A non-coding trans-activating RNA (tracrRNA) mediates the binding of Cas9-crRNA complexes to target loci in type II CRISPR/Cas9 systems [145]. The Cas9-crRNA-tracrRNA complex causes double-stranded breaks (DSBs) in the target-specific gene if the target genetic locus has a protospacer-adjacent motif (PAM) compatible with Cas endonuclease binding as shown in Figure 2. CRISPR/Cas9 functions as a critical component of microbial immune responses in this fashion [146] and the bacterial CRISPR system is reported to be utilized as an experimental disease model that enables genome editing, in turn regulating gene expression levels through CRISPR interference (CRISPRi) and CRISPR activation (CRISPRa) [147]. These genetic perturbations can be further implemented to draw parallel genetic screens to evaluate the functional consequences for human cells. CRISPR screens are specifically stronger when combined with pluripotent stem cell technique, ensuring the sourcing of differentiated cells such as neurons, glial cells, and brain organoids from diseased subjects [34].

The most significant neuropsychiatric disorders are anxiety disorders (AD), schizophrenia (SP), major depressive disorders (MDD), bipolar disorders (BP), autistic spectrum disorders (ASD), and attention deficit hyperactivity disorders (ADHD) [35]. There are many faulty genes responsible for these NPD conditions, (such as AS3MT, CSMD1, ANK3, CNNM2, TENM4, CACNA1C, PPP1R11, CACNB2, NT5C2, DPR1, TCF4, ITIH3, SYNE1, TRIM26, and ZNRD1) and they are mainly expressed in neuronal activity immune regulation, synaptic transmission, and cellular mechanism. Now, the faulty genes are further explored for rectification through CRISPR/CAS9 techniques [147].

It has been seen that DNA methylation is implicated in cognitive performance and retention mechanisms [148,149,150], and many DNA methyltransferases (Dnmts) (Dnmt1, Dnmt2, Dnmt3a, Dnmt3b, and Dnmt3L) have a significant role in CNS development [151]. Dnmt1 and Dnmt3a gene knockout mouse models demonstrated impaired long-term plasticity in the hippocampal CA1 area along with learning and memory problems [152]. Alterations in these genes lead to a smaller hippocampus region and loss of neuronal activity. Both genes have a crucial function in demethylation alteration and are linked with memory storage along with processing dysfunction, resulting in neuron degeneration.

Then, the BDNF protein too has an important role in the development and progression of NPDs as patients with suicidal ideation and psychological illnesses were found with a higher level of BDNF expression [153,154,155]. Moreover the level of BDNF with SLC64 is utilized as a biomarker to diagnose mood disorders in diseased/healthy subjects [156,157], and there are many other genes (NR3C1 and FKBP5) that have also been linked to the early diagnosis of mood disorders [158]. The RELN gene regulates the expression of Reelin protein in GABAergic neurons, which aids in synaptic formation and neurogenesis.

Besides this, Gregorio et al. reported mutations in the PCDH and CTNNA2 genes that lead to schizophrenia. Genome-wide association studies (GWAS) have identified that BDNF, RELN, DRD1, and Dmnt3a are putative targets for the treatment and diagnosis of NPDs [159]. As discussed above, another important factor is a mutation in non-coding regions of the genome, i.e., long non-coding RNAs and microRNAs. CRISPR was used to manipulate non-coding RNAs and silenced many genes, i.e., miR21, miR29a, UCA1, and MALAT [160]. In schizophrenia, DISC1 is one of the major risk factors and scaffold proteins that help in interaction with other proteins required for the dopamine system [161].

Subsequently, Priya et al. (2015) interrupted the DISC1 gene by TALENs and CRISPR/Cas9 in neural cells of the human model, disrupting the gene near the translocation site. The results exhibited decreased DISC1 levels in Wnt signaling with decreased expression of Foxg1 and Tbr2, showing decreased symptoms of schizophrenia [162].

Autism spectrum disorder (ASDs) CHD8 is one of the important genes responsible for regulation of the DNA structure and its mutation, causing the heavy brain, speech delay, characteristic facial features, and other symptoms of autism [163].

One such study was designed to understand the molecular mechanism of CHD8 in ASDs by using CRISPR/Cas9 technology, wherein they knocked out a single copy of CHD8 in iPSCs to mimic the extant LOF (loss of function) in human embryos before neuronal differentiation. With various tools such as transcriptomic and bioinformatic analysis, it was concluded that CHD8 hemizygosity causes the change in expression of many genes in differentiating progenitors of neurons. The results have shown that the differentially expressed genes have GOF (gain of function) for neural development, skeletal development, and β-catenin/Wnt signaling [164]. For monogenic autism spectrum disorder, CRISPR/KO was used for knocking out UBE3A-antisense transcript silencing in mouse models and successfully rescued the phenotype of the mouse [165]. It is indeed very important to check the modified CRISPR’s safety, sensitivity, and specificity before introducing it to humans [166].

Base Editing, CRISPR/Cas9, and gene therapy are contrasted. Cas9, a multi-component protein in the CRISPR/Cas9 system, identifies a typical G-rich PAM at the 3’ end of the target site. Cas9 must be targeted using CRISPR RNA and trans-activating CRISPR RNAs. Blunt ends are produced by the Cas9, which causes a DSB (B). The desired gene is put into the chosen viral vector in gene therapy and then transformed within the target cell. The targeted protein is produced. Cytidine, Adenine, and Histone deaminase coupled with dCas9 are utilized in base editing to target the targeted region.

## 10. Challenges Associated with the Application of CRISPR in Neuropsychiatric Disorders

Despite the encouraging research going on into gene therapy, especially with the inclusion of the very promising CRISPR system, it still may not be easily and immediately translated into a therapeutic tool for neurological disorders. Concerns such as the instant and the highly active response of neurons (originated from stem cells) to any kind of DNA damage in the form of neuronal toxicity or cellular apoptosis are still major hindrances [167]. It is also important to pay attention to the regulation system in the CNS, as gene regulatory mechanics themselves ensure the structural and physiological framework and functional aspects of all the neuronal pathways are kept intact [168,169]. Therefore, the epigenetic mechanism alterations may cause instability in the neural circuital system [170]. A recently published report, in 2020, discussed the possibility of endogenous ion channel upregulation CRISPRa causing a controlled neural spiking rate, thence optimizing the neural network behaviors in epilepsy pathology. Therefore, in this case, CRISPRa could be a suitable, secure, and harmless editing tool for transcription alteration [171].

Another primary concern is passing through the blood-brain barrier (BBB) system, as this physiological barrier holds the cerebral microenvironment very tightly and isolates it completely from other segments of the human body to protect this intricate neural network system. Therefore, a well-targeted approach is required to access the BBB without disrupting its integrity and biological functioning [172]. Many approaches, such as micro dialysis and brain perfusion studies, have not performed well, though nanoparticle-based targeted delivery has shown some positive results [173,174,175].

## 11. Other Methods of Genome Sequencing to Treat NPDs

### 11.1. GWAS (Genome-Wide Association Studies)

In the early 21st century, the genotyping method has made it easy for researchers to perform genotyping arrays by simultaneously assessing one million variants of DNA called SNPs (Single Nucleotide Polymorphisms) [176]. Later on, with the introduction of linkage disequilibrium patterns in the genome, GWAS came into consideration due to captured knowledge about variants in human genomes [112]. According to NIH, GWAS is an observational study set of genomes from genetic variants to check the association of traits with the variants [https://www.genome.gov/genetics-glossary/Genome-Wide-Association-Studies] (accessed on 17 October 2022), making it feasible to address large test sample sizes, and candidate gene approach limitations, and allow unbiased assessment of human genomes. For large sample sets, the significant threshold required is ‘*p* < 5 × 10^−8^’ [177]. Genome-wide association studies have been greatly involved in the identification of risk loci and functional studies. Progress in GWAS leads to the identification of risk factors and variants in NPDs including schizophrenia (SZ), with great success under collaborative efforts by the Psychiatric Genomics Consortium (PGC). The candidate genes with high confidence SZ risk loci are involved in glutamate (GRIN2A, GRM3, GRIA1, SRR), dopamine (DRD2), and calcium channel signaling (CACNA1C, CACNB2, CACNA1L) [178]. 

Equivalently, Rainaid et al. [179] performed analysis of SZ by GWAS-derived genes and linked them to the ischemia–hypoxia response of the brain, wherein they selected the sub-sets of GWAS-based SZ genes and then made the subset of monogenic disorders, multi-omic data, and synaptic proteins. Further, their study analyzed the SZ genes derived from GWAS for the role of HIFs (hypoxia-induced factors) and devised a model for Gene X environment X interactions. The model described the obstetric complication associated with ischemic hypoxia for activating their respective response genes and overlapping of IHR genes with SZ GWAS gene subsets. Their results revealed that any changes in gene expression of ischemic hypoxia may disturb neuronal development, causing a high risk of SZ occurrence. 

Another group of Douglas et al. [180] performed the GWAS of multiplex SZ pedigrees in which they investigated the association of SZ to SNPs and CNV (copy number variants) by sampling 2461 individuals amongst 631 pedigrees, and the polygenic scores of family predicted the status of case-control in the schizophrenia PGC dataset. The results revealed that there were no significant genome-wide associations for SNPs but, on the other hand, the PGC case-controls showed varied genome-wide polygenic scores [180]. 

### 11.2. Whole Exome Sequencing (WES)

Recent decades have witnessed significant advancements in next-generation sequencing (NGS) for genome studies, and amidst them, whole-exome sequencing has come across as an effective and efficient tool. Statistically, in the human genome, only 2% is constituted by exomes (a region in the genome that is composed of exons); however, it holds approximately 85% of all known disease-linked variants, thus making it hugely economical when compared to its counterpart, which is the whole genome sequence [181]. Such technology has a lot of potential as well as vast applications, stretching from discovering and identifying variants to attaining extensive coding region coverage. Further, due to its specificity it produces smaller data sets of around 4–5 GB which makes it easier to arrange and analyze as compared to WGS, which tends to produce data sets of ~90 GB [182]. 

Several researchers have employed this NGS technique to unveil facets of NPDs that correlate to the subject’s genetic predispositions. An elaborate research study was conducted by Cukier et al., 2014, which identified that many variant genes in autism spectrum disorder (ASD) affected patients. Their study involved the WES of 100 ASD patients from 40 unrelated families. They were able to identify many genes which were associated with NPDs other than ASD. Genes such as OFCC1 (Tourette syndrome), SLIT3 (depression), WDR60 (schizophrenia), CLCN2 and PRICKLE1 (epilepsy), and AP4M1 (intellectual disability) do not belong to ASD patients, yet these genes tend to show their presence [183].

Another scientific study was carried out by Halvorsen et al., 2021, using WES for investigating harmful coding variants in OCD subjects. The study encompassed 1313 OCD-affected subjects, the largest cohort to date, including 644 singletons, 587 trios, and 41 quartets of affected subjects. The study identified SLITRK5 and CHD8 as the primary genes exhibiting loss of function mutation and expressed as damaging coding variants. Natively, the SLITRK5 gene produces SLIT & NTRK-like protein 5 that controls the genesis of the excitatory and inhibitory synapse. In a prior study, mice having the SLITRK5 knockout gene were reported to exhibit OCD-like behaviors with raised anxiety and panic levels [184]. On the other hand, after a detailed evaluation of de novo mutations (DNMs), it was determined that the CHD8 gene tends to pose a latent threat in OCD. The CHD8 gene originally functions as transcriptional regulation of neuronal development; however, due to loss of function mutation in the CHD8 gene, it becomes involved in ASD as well as in other NPDs other than OCD [185].

Along similar lines, whole-exome sequencing was performed aimed at exploring and identifying novel coding variants implicated in a broad spectrum of neuropsychiatric syndromes, Ganesh et al., 2019 [186]. The study included 33 diseased subjects who were screened from eight multiplex families along with 33 healthy subjects. Results from the study highlighted novel gene variants identified (chr3:1222423522 & GRch37) in the PARP14 gene that is involved in post-traumatic stress disorder (PTSD), attention deficit hyperactivity disorder (ADHA), and major depressive disorder (MDD). Variant rs148371256 in the NRG2 gene is reported to be implicated in the synthesis as well as the maturation of GABAergic synapses. Besides these variants, the rs534059912 variant was also identified in the GOLM1 gene that influences prefrontal cortical volume in subjects affected with AD [187,188].

## 12. Discussion and Conclusions

The emergence of CRISPR-based genome editing as a viable therapeutic option for correcting the base pathogenesis of various disorders has been recognized as one of the most promising potential therapeutic tools. Mutations in genes of interest can be generated efficiently using CRISPR-mediated NHEJ (non-homologous end-joining), minimizing the time taken for gene discovery and exploration of its mechanisms [189]. The transfer of CRISPR constituents (gRNA, tracrRNA, and Cas9) to cells or model organisms is an effective path that can be successfully attained by mRNA transfection or distribution through the use of adeno-associated viruses (AAVs) [190]. Likewise, the recently developed Cre-inducible Cas9 mouse enabled the transmission of a single AAV that included the combination of gRNA, tracrRNA, and Cre. With relative ease, this prototype can remove the desired genes in distinct groups of neurons in animal models with around 80% reliability knockout in their targeted gene [191]. The potential of CRISPR to target multiple genes at the same time is notably advantageous when we explore the role of gene interplay in NPDs. Furthermore, when used in conjunction with uniquely engineered DNA templates, CRISPR-mediated HDR can imitate alleles that affect the likelihood of NPDs or, eventually, improvise the risk-modifying alleles [192]. The most awaited application is in CNS disorders and for NPDs. These NPDs are now very keenly observed for their genetic mechanism and further expanded with more inclusions of genes to be edited. The main concern is related to the safety of the editing performed, as still more exploration and precision validation is needed in this direction. The other important concern with the delivery system of the gene material at the targeted site is that it has to bypass or cross the BBB, so it should be therapeutically designed such that apart from impregnating through the physiological barriers it should be non-immunogenic and non-toxic to the entire cerebral atmosphere. Moreover, in this case, suppression of immunogenic responses via gene therapy is not recommended as it may pose higher health hazards. Subsequently, there are other related issues as well that need consideration, such as the cost of gene therapy and ethical concerns related to attaining superior phenotypic characteristics. However, overall we can characterize this technique in the near future as having the full potential to turn into an appropriate answer for most diseases.

With time, it has been realized that most psychiatric disorders are directly or indirectly related to alterations in epigenetic or gene regulatory events, and with the inclusion of CRISPR/Cas9 targeted editing mechanism-type tools, the possibility to correct these genetic errors seems feasible. The CCS-induced NPDs are no different, and can be very well catered to with such options. This technique has also led to the exploration of functional genomics in CNS diseases and the regulating of human genome sequences. New genome editing technologies, however, are being created swiftly and will greatly increase targeting rates while minimizing off-target mutation impacts [193]. The development of non-human primate and big animal models of neuropsychiatric disorders will be facilitated by CRISPR/Ca9 technology, which will also improve our understanding of the pathophysiology of these significant diseases [33].

## Figures and Tables

**Table 1:** Associated gene loci in various neuropsychiatric disorders.

S.No.	Name of the NPD	Genes/Genetic Locus Associated	References
1	Schizophrenia	MTHFR, CGI3L1, DISC1. DISC2, SYN2, DRD3, RTN4R, DAOA, HTR2A, AKT1, C4A, APOL2. APOL4, PRODH, NRG, SHANK3, NRXN1, SLC1A1, RBM12	[16,115,116,117,118]
2	Autism	CNTNAP2, SLC9A9, SHANK2, CHD8, EIF4E, BLGN1, NLGN3, NLGN4X, MECP2, PTCHD1, RPL10, TMLHE	[16,119,120,121,122,123]
3	Fragile X Syndrome	FMR1	[124,125]
4	Epilepsy/Seizures	CACNA1H, CASR, CACNB4, GABRD, CLCN2, SLC2A1, GABRA1, SLC12A5, RORB, KCNMA1	[126,127,128,129]
5	Parkinson’s disease (PD)	SNCA, Parkin, UCHL1, PINK1, DJ1, LRRK2, ATP13A2, GIGYF2, HTRA2, PLA2G6, FBX07, VPS35, EIF4G1, DNAJC6, CHCHD2, VPS13C, PSAP, NR4A2, MAPT, PARK2, PARK6, PARK8	[130,131,132,133,134]

## Data Availability

Not applicable.
